# Cutaneous Larva Migrans on the Palm: An Unusual Site with Dermoscopic Correlation

**DOI:** 10.4269/ajtmh.26-0266

**Published:** 2026-07-14

**Authors:** Sejal Jain, Keshavamurthy Vinay

**Affiliations:** Department of Dermatology, Venereology and Leprology, Postgraduate Institute of Medical Education and Research, Chandigarh, India

## INTRODUCTION

A 45-year-old female agricultural worker presented to the dermatology department for evaluation of a linear skin rash on her left palm of 3 months’ duration. The lesion progressed by a few millimeters every day and was associated with intense itching, disturbing her sleep. She had a history of harvesting flowers prior to the onset of lesions.

On cutaneous examination, there was a single serpiginous tract extending across the thenar eminence (Figure 1A). It was orangish-yellow distally and erythematous at the proximal end. The leading edge showed a small, crusted point corresponding to the larval entry site. Dermoscopy revealed the reddish-brown whiplash-like tunnel with surrounding erythema and scattered red dots and clods (Figure 1B). Focal yellowish-brown scaling and a terminal crusted point corresponding to the larval entry site was seen. A clinical and dermoscopic diagnosis of cutaneous larva migrans (CLM) was made.

**Figure 1.**
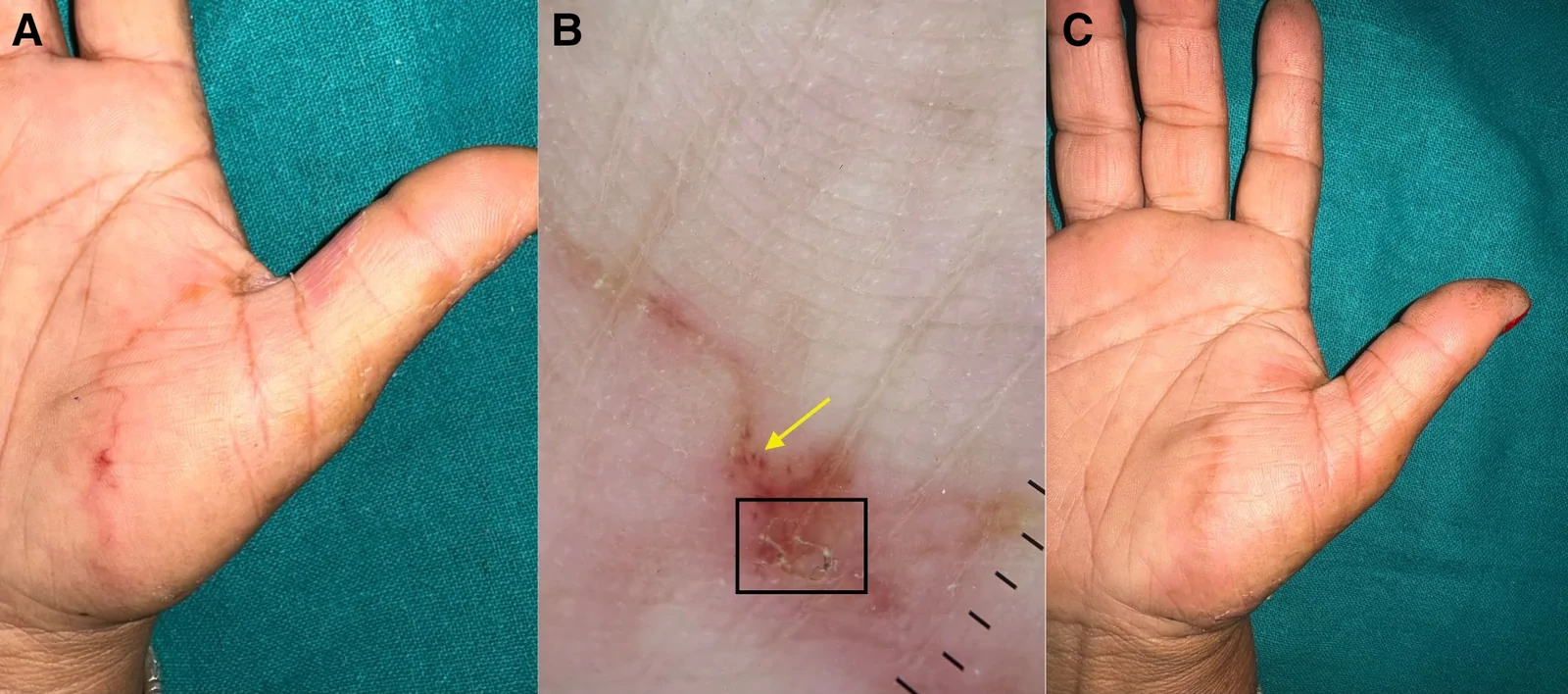
(**A**) Single serpiginous tract extending across the thenar eminence. (**B**) Dermoscopy revealing a reddish-brown whiplash-like tunnel with peripheral erythema, scattered red dots (yellow arrow), and a crusted terminal entry point (black box). (Dermlite DL4, polarised mode, 10×). (**C**) Post treatment clinical image showing resolution of signs of inflammation (erythema, hemorrhagic crusting) following treatment.

Also known as creeping eruption, CLM is a neglected zoonotic tropical disease caused by the accidental percutaneous penetration of animal hookworm larvae, most commonly *Ancylostoma braziliense* and *A. caninum*.^1^ Although typical sites include the feet, buttocks, and thighs, palmar involvement is rare. Dermoscopy serves as a rapid, noninvasive adjunct for confirmation.^2^ Our patient was treated with a single oral dose of ivermectin (200 *µ*g/kg), resulting in complete resolution of the lesion and pruritus within 5 days. Dermoscopically, there was disappearance of the red dots, clods and erythema; the remnant tunnel was visible (Figure 1C).

Recognition of CLM in atypical locations is crucial to avoid misdiagnosis as scabies or phytophotodermatitis.
